# Oral Immunization with Recombinant *Lactobacillus acidophilus* Expressing espA-Tir-M Confers Protection against Enterohemorrhagic *Escherichia coli* O157:H7 Challenge in Mice

**DOI:** 10.3389/fmicb.2017.00417

**Published:** 2017-03-15

**Authors:** Ruqin Lin, Yiduo Zhang, Beiguo Long, Yawen Li, Yuhua Wu, Siqin Duan, Bo Zhu, Xianbo Wu, Hongying Fan

**Affiliations:** ^1^Guangdong Provincial Key Laboratory of Tropical Disease Research, School of Public Health, Southern Medical UniversityGuangzhou, China; ^2^The First School of Clinical Medicine, Southern Medical UniversityGuangzhou, China

**Keywords:** *Escherichia coli* O157:H7, *Lactobacillus acidophilus*, live vector vaccine, A/E lesions, probiotics

## Abstract

Enterohemorrhagic *Escherichia coli* O157:H7 (EHEC O157:H7) causes hemorrhagic colitis and the formation of characteristic attaching and effacing (A/E) lesions in humans. Given the severe sequelae of EHEC O157:H7 infection, it is critical to develop effective vaccines for human use. However, for achieving this goal many hurdles need to be addressed, such as the type or subset of antigens, adjuvant, and the delivery route. We developed a candidate vaccine by inserting the bivalent antigen espA-Tir-M composed of espA and the Tir central domain into *Lactobacillus acidophilus*. The recombinant *L. acidophilus* (LA-ET) was safe in a cell model and excluded EHEC O157:H7 from LoVo cells at rates of nearly 94 and 60% in exclusion and competition assays, respectively. LA-ET inhibited the induction of A/E lesions by EHEC O157:H7 cells *in vitro*. Oral immunization with LA-ET induced higher levels of specific mucosal and systemic antibody responses in mice. Moreover, LA-ET enhanced interferon-γ and interleukin-4 and -10 production, which was associated with mixed helper T (Th1/Th2) cell responses, and protected against EHEC O157:H7 colonization and infection in mice at a rate of 80%. Histopathological analyses revealed that orally administered LA-ET reduced or inhibited A/E lesions and toxin-induced systemic injury. These findings demonstrate that LA-ET induces both humoral and cellular immune responses in mice and is therefore a promising vaccine against EHEC O157:H7 infection.

## Introduction

Enterohemorrhagic *Escherichia coli* O157:H7 (EHEC O157:H7) is a zoonotic enteric pathogen that causes hemorrhagic colitis and hemolytic uremic syndrome (HUS) in humans (Remis et al., 1984; Armstrong et al., 1996). EHEC O157:H7 can intimately adhere to intestinal epithelial cells and form characteristic attaching and effacing (A/E) lesions, and antibiotic treatment can potentially increase HUS risk (Besser et al., 2001; Wong et al., 2012). It is presumed that a vaccine that prevents EHEC from colonizing the intestinal tract would be the most effective strategy for preventing infection (Mayr et al., 2005). Currently, two vaccines are used for the same purpose in cattle, but neither has been approved for humans (Larrie-Bagha et al., 2013). For achieving this goal many hurdles need to be addressed, such as the type or subset of antigens, adjuvant, and the delivery route.

The virulence of EHEC O157:H7 can be largely attributed to its toxins, type III secretion system (T3SS) proteins, and surface fimbrial and afimbrial adhesions (Garcia-Angulo et al., 2013). The T3SS delivers espA, espB, and espD directly to the host cells, which are essential for signal transduction and A/E lesion formation (Frankel et al., 1998). The major component, espA forms a bridge to host cells for direct delivery of other virulence factors (Li et al., 2000). Tir is one of the first proteins translocated during infection and functions as a receptor for the EHEC outer membrane protein, intimin (DeVinney et al., 1999). The T3SS proteins, espA and Tir thus play key roles in EHEC adhesion, pedestal formation, and pathogenicity. They may also serve as immunogens for the disruption of bacterial-host cell interaction and prevent the formation of A/E lesions.

Lactic acid bacteria (LAB) such as *Lactobacillus acidophilus (L. acidophilus), Lactococcus lactis*, and *Bifidobacterium* have been given a “generally regarded as safe” (GRAS) designation and are widely used in human food production. Additionally, LAB’s are considered as a useful tool for the development of novel oral vectors and have largely replaced attenuated pathogens with mucosal delivery strategies (Bermudez-Humaran et al., 2011). Moreover, LAB can also be used as delivery vectors for subunit vaccines, which circumvents costly purification processes (Wells, 2011). Vaccines employing a mucosal delivery system can elicit both antigen-specific secretory immunoglobulin A (sIgA) and effective systemic immune responses. The relatively low cost, ease of administration, and degree of mucosal tissue protection make LAB more advantageous as vaccine delivery vectors (Rosales-Mendoza et al., 2016). *Lactococcus lactis is* most often used for EHEC and other pathogen vaccines. *Lactococcus lactis*-expressing EspB induces protective immunity against EHEC O157:H7 and recombinant *Lactococcus lactis* induces protection against *C. difficile* (Ahmed et al., 2014; Guo et al., 2015). However, there are few reports describing the use of *L. acidophilus* as a vaccine delivery vector that has been approved for use in infant and baby food. *L. acidophilus* is particularly promising as an oral vaccine vector because it is acid- and bile-tolerant, can express mucus-binding proteins that enable association with intestinal mucosa and regulates immature dendritic and T cell functions (Konstantinov et al., 2008). To date, there have been no studies of recombinant *L. acidophilus* producing the EHEC O157:H7 protective antigen as a candidate vaccine. In this study, we developed a candidate EHEC O157:H7 vaccine by inserting the bivalent antigen espA-Tir-M (ET), which is composed of espA and the Tir central domain (Tir-M), into *L. acidophilus*. We evaluated the specific immune responses elicited in mice and protection against EHEC O157:H7 challenge both *in vitro* and *in vivo*.

## Materials and Methods

### Bacteria, Plasmids, and Cells

*Lactobacillus acidophilus* ATCC4356 (American Type Culture Collection, Manassas, VA, USA) was cultured in de Man, Rogosa, and Sharpe (MRS) agar or broth at 37°C in an atmosphere of 5% CO_2_. EHEC O157:H7 strain EDL933 (our laboratory stock) was grown at 37°C in Luria–Bertani (LB) broth supplemented with 5 g/l streptomycin. The pMG36e plasmid (our laboratory stock) was maintained in DH5α cells and cultured in LB broth with 200 μg/ml erythromycin, and pMG36e-based plasmids were maintained in MRS agar or broth with 0.1 μg/ml erythromycin. The LoVo human colonic cancer cell line (our laboratory stock) was cultured in Roswell Park Memorial Institute (RPMI) 1640 medium containing 10% fetal bovine serum (FBS) at 37°C in a humidified atmosphere of 5% CO_2_.

### Construction of Recombinant *L. acidophilus* Strain

To construct the recombinant plasmid pMG36e-espA-Tir-M, the espA-Tir-M (ET) fragment was amplified by overlap extension PCR from espA (GenBank accession no. KJ549678.1) and Tir-M (GenBank accession no. NC002655.2). EspA and Tir-M fragments were joined with a linker (GGA GGC GGA AGT GGA GGA GGT AGC). Both espA and Tir-M were PCR-amplified from *E. coli* O157:H7 EDL933 using primer pairs with the following sequences: P1 (forward), 5′-A AAA CTGCAG GAT GGA TAC ATC AAA TGC A- 3′ and P2 (reverse), 5′-GCT ACC TCC TCC ACT TCC GCC TCC TTT ACC AAG GGA TAT TGC TG-3′ for espA; P3 (forward), 5′-GGA GGC GGA AGT GGA GGA GGT AGC AGC CCA ACC ACG ACC GAC-3′ and P4 (reverse), 5′-CCC AAGCTT TTA GGC TTG CTG TTT GGC CTC TT-3′ for Tir-M; and P1/P4 for espA-Tir M. Restriction enzyme sites (*Pst*I and *Hind*III) are underlined. The fusion gene espA-Tir-M was inserted into plasmid pMG36e.

The recombinant plasmid pMG36e-espA-Tir-M was transformed into *L. acidophilus* cells according to the manufacturer’s instructions for the expression system in *Lactococcus lactis* (MoBiTec, Germany) with appropriate adjustment. Briefly, cells were cultured in 4 ml of MRS broth with 0.05% cysteine-HCl medium at 37°C for 48 h. When the optical density at 600 nm (OD_600_) reached 0.6, 4 ml of culture was diluted in 100 ml of MRS broth with 0.5 M sucrose and 0.05% cysteine-HCl, followed by incubation for approximately 24 h until the OD_600_ was 0.8. The culture was cooled for 10 min and centrifuged at 5,000 rpm for 20 min at 4°C, then washed twice in 20 ml of 0.5 M sucrose buffer. The cells were re-suspended in 5 ml of pre-cooled transformation buffer composed of 10 mM ammonium and 0.5 M sucrose (pH = 6.0), then centrifuged at 5,000 rpm for 20 min at 4°C and re-suspended in 400 μl of transformation buffer. The recombinant plasmid was transformed into *L. acidophilus* cells by electroporation using a Pulse Controller apparatus (Bio-Rad, Hercules, CA, USA) at 2.5 kV and 25 μF. Transformed bacteria were re-suspended in MRS broth and cultured at 37°C for 1 h, and were plated on MRS agar (1.5%, w/v) with 0.1 μg/ml erythromycin at 37°C for 48 h. Positive colonies of transformed bacteria were identified by PCR-amplifying the target gene (espA-Tir-M) and sequencing. The empty vector pMG36e transformed into *L. acidophilus* cells (LA) served as a control.

### Sodium Dodecyl Sulfate Polyacrylamide Gel Electrophoresis (SDS-PAGE) and Western Blot Analysis

The recombinant *L. acidophilus* (LA-ET) was cultured in MRS broth with 0.1 μg/ml erythromycin at 37°C for 24 h. The supernatant was precipitated with trichloroacetic acid followed by two washes with acetone. A 20 μl volume of sterile distilled water was added to dissolve the supernatant proteins, which were separated by 12% (w/v) sodium dodecyl sulfate polyacrylamide gel electrophoresis (SDS-PAGE) and analyzed by western blotting using an anti-EHEC O157:H7 polyclonal antibody (EterLife, Birmingham, UK) according to the manufacturer’s instructions.

### Cytotoxicity Analysis of LA-ET

LA-ET or LA at a multiplicity of infection (MOI) of 500:1 was incubated with LoVo cells for 12 h, after which these cells were harvested and washed with PBS. The cells were re-suspended in 500 μl of binding buffer containing 5 μl of FITC-Annexin V and 5 μl of PI. The cells were gently vortexed and incubated for 15 min at room temperature in the dark. Finally, the cells were detected using flow cytometry according to the manufacturer’s instructions.

### Inhibition of EHEC O157:H7 by Colonized LoVo Cells

Inhibition of EHEC O157:H7 by LA-ET by the colonized LoVo cells was evaluated using the following assays as previously described (Satish Kumar et al., 2011). Briefly, 1 × 10^5^ LoVo cells per well were cultured in 24-well plates. For the exclusion assay, the LoVo cells were pre-inoculated with LA-ET or LA (MOI = 500:1) for 90 min and then, EHEC O157:H7 (MOI = 100:1) was added for co-incubation for 90 min at 37°C. For the competition assay, LA-ET or LA and EHEC O157:H7 were added to LoVo cells, followed by incubation at 37°C for 3 h. LoVo cells inoculated with EHEC O157:H7 (MOI = 100: 1) for 90 min served as a positive control. To measure the total number of adherent EHEC O157:H7 bacteria, cells were lysed and the cell suspension was plated in triplicate on LB agar and incubated for 24 h at 37°C, after which the number of colony-forming units (CFUs) was counted. Each experiment was repeated three times. Results were calculated as a relative adhesion percentage [100 × (number of adherent bacteria in the experimental group)/(number of adherent bacteria in the positive control group)].

### LA-ET Inhibits EHEC O157:H7-Induced Attaching and Effacing Lesions

Fluorescence actin staining (FAS) was performed to detect the formation of A/E lesions. Briefly, LoVo cells were pre-incubated with LA-ET (MOI = 500:1) for 3 h at 37°C and then EHEC O157:H7 (MOI = 100:1) cells were added for co-incubation for 3 h at 37°C in 5% CO_2_. After incubation, cells were washed three times with PBS (pH 7.4) and fixed in 4% paraformaldehyde in PBS (pH 7.0) for 10 min. Following a 30 s wash in PBS, cells were permeabilized in 0.5% Triton X-100 in PBS for 5 min. The cells were washed once and then 200 μl of 100 nM rhodamine phalloidin (Cytoskeleton, USA) was added and incubated in the dark for 30 min. The cells were washed three times and the DNA was counterstained for 30 s with 200 μl of 100 nM DAPI in PBS. The samples were examined with a confocal laser scanning microscope.

### Oral Immunization of Mice with Recombinant *L. acidophilus* Strains

Specific pathogen-free 6-week-old female BALB/c mice were obtained from the Southern Medical University Laboratory Animal Center (Guangzhou, China). All experimental and animal handling procedures were approved by the Institution Animal Care Committee of Southern Medical University (permit no. 44002100006397) and were performed in accordance with the approved relevant guidelines. The mice were sacrificed by cervical dislocation at the end of the experiment. LA-ET strains were grown as described above, then collected and washed twice with sterile PBS. Mice were randomly divided into the following three groups (*n* = 15): PBS, LA and LA-ET. The PBS group was orally administered with 100 μl sterile PBS; the LA group was orally administered with 1 × 10^9^ CFU LA; and the LA-ET group was orally administered with 1 × 10^9^ CFU LA-ET. All mice were immunized on days 0, 3, 7, 10, 21, and 24 (the day of first immunization regarded as 0 day) and serum samples were collected on days 0, 7, 21, and 35 and stored at -80°C until use. Fecal extracts were collected on day 35 for sIgA measurement by enzyme-linked immunosorbent assay (ELISA) as previously described (Amani et al., 2010).

### Determination of Antibody and Cytokine Levels by ELISA

Total serum IgG (total IgG) and fecal IgA were measured by indirect ELISA as previously described (Gao et al., 2009). ELISA plates were coated with 100 μl of 10 μg/ml purified espA-Tir-M protein (our laboratory stock). Serum and fecal extracts were prepared as described above and diluted 1:50 and 1:10, respectively. In addition, 5 μl serum (collected on day 34) was assayed for murine interferon (IFN)-γ and interleukin (IL)-4 and IL-10 by quantitative ELISA using a mouse ELISA kit (Elabscience, Hubei, China) according to the manufacturer’s instructions. ELISA results were obtained by measuring the OD_450_ using an EL9800 ELISA microplate reader (BioTek, Winooski, VT, USA).

### Survival Analysis of Immunized Mice

At 10 days after the last immunization, mice in each group were randomly divided into two subgroups, of which one subgroup (*n* = 10) received streptomycin (5 g/l) in the drinking water from 3 days before infection until the end of the experiment to clear intestinal flora and to enhance EHEC O157:H7 colonization (Wadolkowski et al., 1990), whereas the other subgroup (*n* = 5) was not treated with streptomycin. Mice were challenged by oral inoculation of 1 × 10^10^ CFU of EHEC O157:H7 in PBS. Fecal shedding of EHEC O157:H7 was monitored at 2-day intervals as previously described (Amani et al., 2010). The number of surviving mice in each group was recorded daily. All remaining mice were sacrificed on day 15 post-challenge.

### Histopathology

Mouse tissues were fixed by immersion in 10% neutral formalin, embedded in paraffin, and cut into sections that were stained with hematoxylin and eosin and was further microscopically evaluated by two expert pathologists who were blinded to the experimental groups.

### Statistical Analysis

Statistical analysis was performed using SPSS v.21.0 software. Differences in antibody and cytokine levels were evaluated by one-way analysis of variance with the least significant difference test. The duration of fecal shedding among groups were evaluated by one-factor repeated measures ANOVA analysis. The number of adherent bacteria was analyzed using Student’s *t*-test. *P* < 0.05 was considered statistically significant.

## Results

### LA-ET Expressing espA-Tir-M Fusion Protein in the Culture Supernatant

**FIGURE 1:**
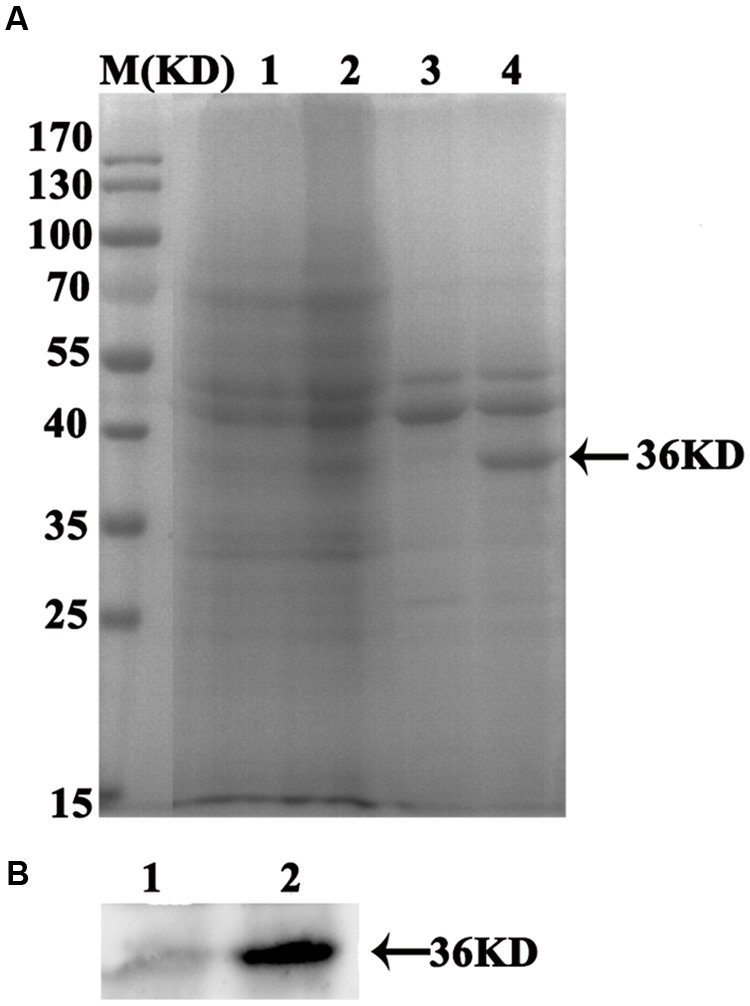
**Sodium dodecyl sulfate polyacrylamide gel electrophoresis (SDS-PAGE) and western blot analysis of proteins released by LA-ET in the supernatant. (A)** SDS-PAGE of LA-ET; M represents the molecular size marker in kDa (Bio-Rad); Lane 1, supernatant of LA lysate; Lane 2, supernatant of LA-ET lysate; Lane 3, culture supernatant of LA; Lane 4, culture supernatant of LA-ET; **(B)** western blot analysis of LA-ET; Lane 1, culture supernatant of LA. Lane 2, culture supernatant of LA-ET.

The espA-Tir-M fusion gene was amplified by overlap extension PCR, yielding the expected 816-bp product, which was visualized by agarose gel electrophoresis (data not shown). The fragment was cloned into the pMG36e vector, and the recombinant pMG36e-espA-Tir-M plasmid was transformed into *L. acidophilus* cells. The supernatant proteins, LA-ET or LA were analyzed by SDS-PAGE and western blotting. A 36-kDa band was observed in the culture supernatant of the LA-ET lysate but not in the supernatant of the LA lysate (**Figure 1A**), which indicates that the LA-ET-expressed espA-Tir-M fusion protein was mainly a secreted protein. The secretion of espA-Tir-M was confirmed by reaction with the anti-EHEC O157:H7 polyclonal antibody with western blotting (**Figure 1B**).

### LA-ET Was Safe in a Cell Model

**FIGURE 2:**
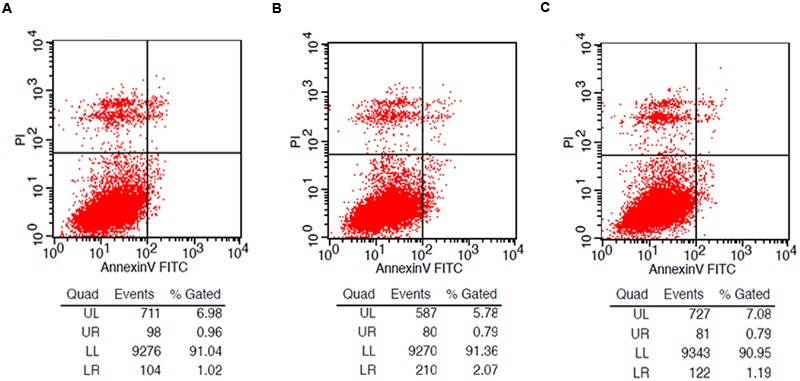
**Flow cytometry to determine the cytotoxicity of LA-ET. (A)** Normal LoVo cells as control; **(B)** LoVo cells incubated with LA; **(C)** LoVo cells incubated with LA-ET.

Comparisons of the cytotoxic effect in LoVo cells showed no obvious difference in the LA-ET group compared to the normal group and LA group (**Figure 2**).

### Inhibition of EHEC O157:H7 Colonization and A/E Lesion Formation in LoVo Cells

**FIGURE 3:**
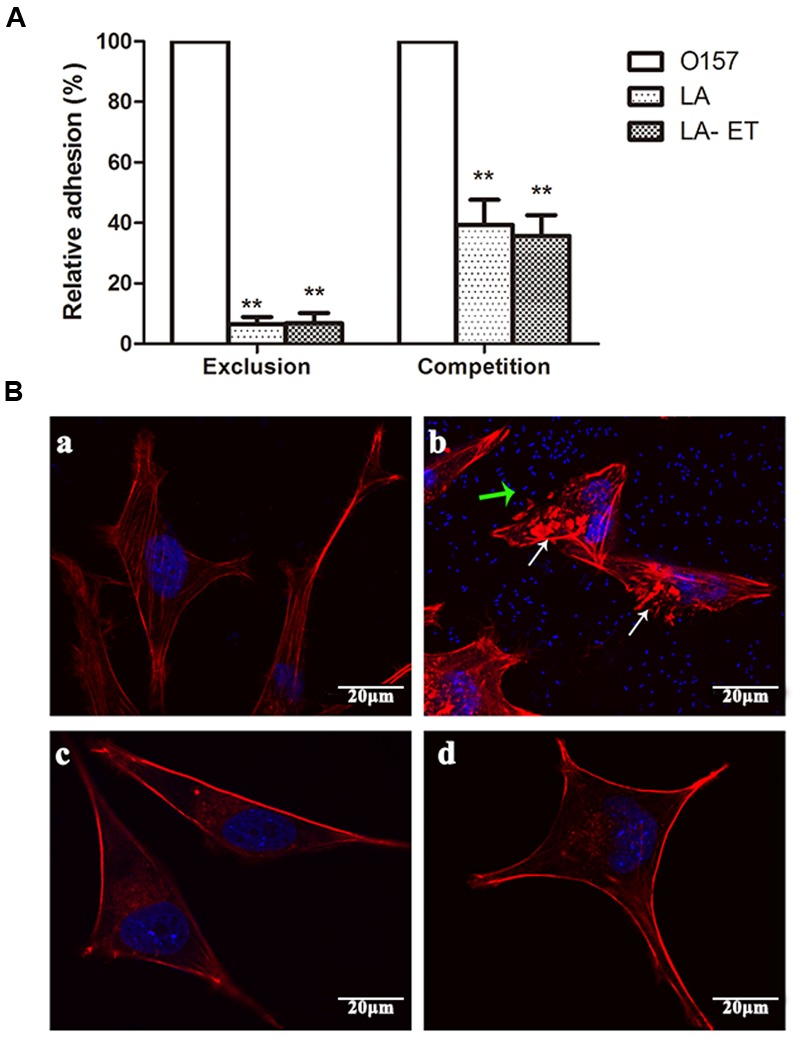
**Inhibition of EHEC O157:H7 colonization and A/E lesion formation in LoVo cells. (A)** Exclusion and competition adhesion inhibition assays in LoVo cells. Results are expressed as relative adhesion relative to the control without LA or LA-et. Data are shown as a percentage ± SD (*n* = 9). ^∗∗^*P* < 0.001 (one-way analysis of variance with least significant difference test). **(B)** FAS detected A/E lesion formation. **(a)** Normal LoVo cell; **(b)** LoVo cells were incubated with EHEC O157:H7 (MOI = 100:1) for 3 h; White arrows show the A/E lesion and green arrows show EHEC O157:H7; **(c)** LoVo cells were incubated with LA-ET (MOI = 500:1) for 3 h; **(d)** LoVo cells were pre-incubated with LA-ET (MOI = 500:1) for 3 h and then EHEC O157:H7 (MOI = 100:1) was added for co-incubation for 3 h.

The ability of LA-ET to interfere with EHEC O157:H7 adhesion to LoVo intestinal epithelial cells was investigated using exclusion and competition assays. For the former, both LA-ET and LA excluded EHEC O157 from LoVo cells at a rate of almost 94% (*P* = 0.000). For the latter, the relative adhesion was 35.8% ± 6.83% for LA-ET and 39.4% ± 8.26% for LA (*P* = 0.501), and both LA-ET and LA excluded EHEC O157 from LoVo cells by about 60% (*P* = 0.000) (**Figure 3A**).

Fluorescent-labeled phalloidin staining of F-actin in LoVo intestinal epithelial cells showed that EHEC O157:H7 challenge of LoVo cells induced bacterial attachment to cells (green arrows), effaced microvilli, and recruited F-actin into pedestals to form A/E lesions (white arrows) (**Figure 3Bb**). By contrast, cells pretreated with the LA-ET no longer demonstrated EHEC O157:H7 adhesion and A/E lesions (**Figure 3Bd**). The LoVo cells (**Figure 3Ba**) and LoVo cells incubated with LA-ET (**Figure 3Bc**) were normal.

### Serum IgG and Fecal sIgA Levels

**FIGURE 4:**
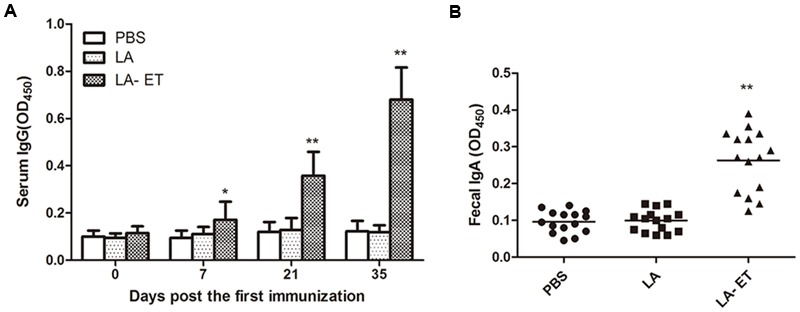
**Detection of specific IgG in serum and sIgA in fecal samples.** Antibody levels were determined by ELISA. The fusion protein espA-Tir-M was used as a coating antigen. Horseradish peroxidase-labeled anti-mouse IgG or IgA was used as the test antibody. **(A)** Serum IgG levels were determined 0, 7, 21, and 35 days after the first immunization. **(B)** sIgA antibody in fecal samples collected on day 35 after the first immunization. Data are shown as the mean ± SD (*n* = 15). ^∗^*P* < 0.05, ^∗∗^*P* < 0.001 (one-way analysis of variance with least significant difference test).

Significant IgG responses were elicited in mice immunized with LA-ET as compared to controls (**Figure 4A**). In addition, serum antibody titer increased after each immunization with LA-ET (0.24 ± 0.09 on day 7, 0.35 ± 0.10 on day 21, and 0.68 ± 0.14 on day 35), reaching a peak on day 35 (*P* = 0.000). sIgA was also measured in fecal samples (**Figure 4B**). Consistent with systemic antibody responses, sIgA levels were increased in mice immunized with LA-ET (0.26 ± 0.08) as compared to control mice (*P* = 0.000).

### IL-4, IL-10, and IFN-γ Levels in Immunized Mice

**FIGURE 5:**
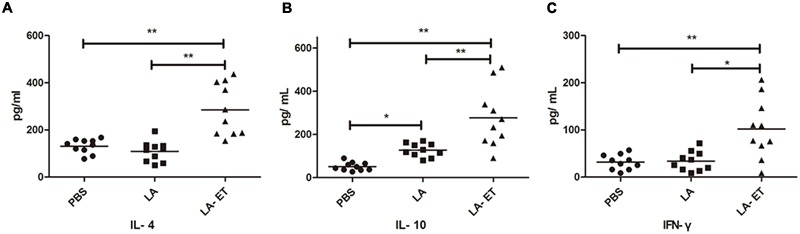
**Serum levels of IL-4, IL-10, and IFN-γ.** Cytokines were detected in serum collected 35 days after the first immunization. **(A)** The serum level of IL-4. **(B)** The serum level of IL-10. **(C)** The serum level of IFN-γ. Data are reported as the mean ± SD (*n* = 10). ^∗^*P* < 0.05, ^∗∗^*P* < 0.001 (one-way analysis of variance with least significant difference test).

Cytokines play an important role as part of the immune response against infections. Serum IL-4 concentrations were significantly higher in the LA-ET group (285.2 ± 110.3 vs. PBS, *P* = 0.000; vs. LA, *P* = 0.000) (**Figure 5A**). Serum IL-10 level was higher in the LA-ET (276.4 ± 138.1, *P* = 0.000) and LA (127.8 ± 31.1; *P* = 0.048) groups than in the PBS group (51.4 ± 19.5) (**Figure 5B**). Moreover, the serum IFN-γ level was higher in the LA-ET group (102.2 ± 63.0) than in the control LA (34.0 ± 20.9, *P* = 0.001) and PBS (31.9 ± 16.0, *P* = 0.000) groups (**Figure 5C**).

### Immunization with LA-ET Protects Mice against EHEC O157:H7 Colonization

**FIGURE 6:**
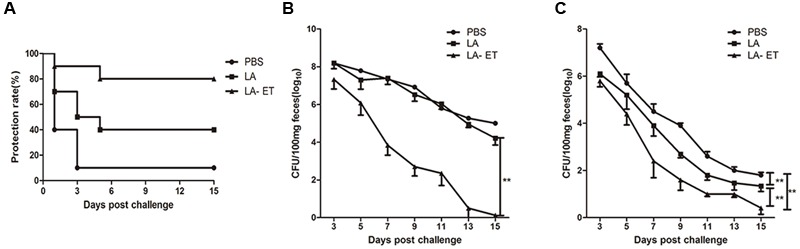
**Survival analysis of immunized mice. (A)** Protection rates in EHEC O157:H7-challenged mice. The number of mice that died was monitored for 15 days post-infection. **(B,C)** Changes in fecal shedding of EHEC O157:H7 in mice. Mice (*n* = 10) were orally challenged with 10^10^ CFU EHEC O157:H7 either under oral streptomycin treatment condition (*n* = 10) **(B)** or without streptomycin treatment (*n* = 5) **(C)** after the last immunization and fecal shedding was monitored for 15 days. The limit of detection for plating was 100 CFU/100 mg feces. Data represent the mean ± SD. Data points in rectangles indicate a significant difference between groups. ^∗∗^*P* < 0.001 (one-factor repeated measures ANOVA analysis).

The protection efficiency of LA-ET was evaluated based on survival, fecal shedding, and histopathology. Within the streptomycin-treated immunization groups, 90% of mice were susceptible to EHEC O157:H7 challenge and died between days 1 and 3 post-infection in the PBS control group (**Figure 6A**), whereas 60% of mice in the LA group died between days 1 and 5. Clinical symptoms included weight loss, hunched posture and lumbering gait. The post-infection survival data revealed that the protection rate was 80% in LA-ET.

In addition, the duration of fecal shedding shown by the LA-ET group was significantly shorter than that in the LA group (*P* = 0.000); one vaccinated mouse had stopped shedding on day 9, and all mice were negative by day 13. In contrast, control mice shed bacteria throughout the 15-day time course (**Figure 6B**).

No mice died in the non-streptomycin-treated immunization groups when challenged with EHEC O157:H7. Comparisons of the durations of EHEC O157:H7 fecal shedding showed a significantly shorter duration in the LA-ET group (8.2 ± 0.89 days) compared to the LA and PBS groups (11.4 ± 0.89 and 13.4 ± 0.89 days, respectively) (*P* = 0.000). Additionally, the shorter duration of fecal shedding shown by the LA group in comparison to the PBS group was significant (*P* = 0.007) (**Figure 6C**).

### Histopathological Findings

**FIGURE 7:**
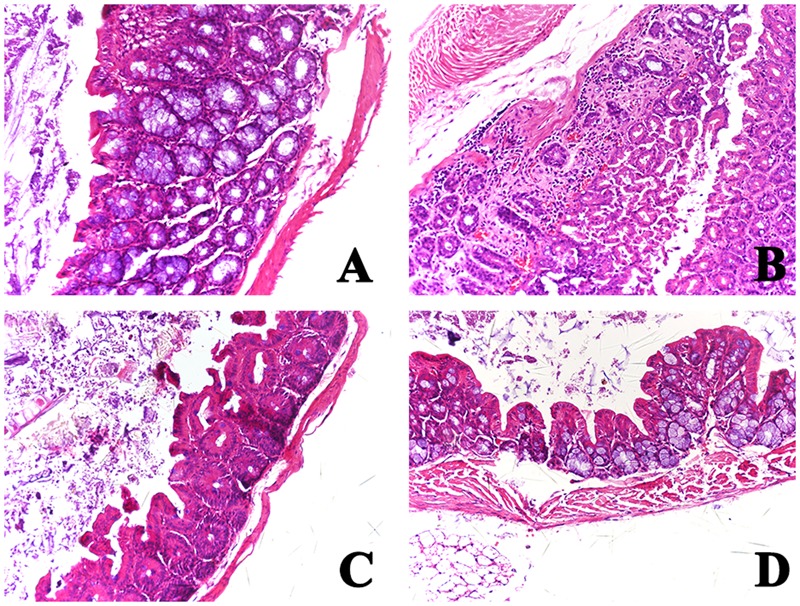
**Histopathological analysis of the colon of immunized mice challenged with EHEC O157:H7.** Tissue was obtained from mice sacrificed 15 days after infection. Sections of paraffin-embedded tissue were stained with hematoxylin and eosin and examined by light microscopy. **(A)** Normal mice; **(B)** PBS group; **(C)** LA group; and **(D)** mice immunized with LA-ET group.

The intestine was dissected out from the mice sacrificed 15 days after challenge. The pathological changes of non-immunized infected mice included major intestinal injury (epithelial cell necrosis) accompanied by damage to the kidney (hyperemia of mesenchyme capillaries), liver (mesenchyme hyperemia), and spleen (splenic sinusoid dilation and ecchymosis) (data not shown). However, the colon of immunized mice infected with EHEC O157:H7 appeared normal, and these animals produced well-formed stools with no obvious mucosal thickening, similar to non-infected mice (**Figure 7**).

## Discussion

Enterohemorrhagic *Escherichia coli* O157:H7 is a major cause of gastroenteritis. Most infected individuals develop HUS, which is prevalent in children (Pennington, 2010). EHEC O157:H7 adheres to host epithelial cells as the first step of the infection process, leading to pedestal formation and effacement of microvilli, which characterize the A/E lesions. Hence, inhibiting pathogen adhesion to epithelial cells may prevent colonization and limit opportunistic infection (Johnson-Henry et al., 2007). It has been suggested that probiotics are effective in preventing adhesion and invasion of enteric pathogens (Dini et al., 2016). Probiotics may reduce intestinal infections by competing with pathogens for binding sites on the intestinal wall as well as for nutrients, and also by producing antibacterial compounds and lactic acid, or via immunomodulation (Roselli et al., 2006; Delcenserie et al., 2008; Amara and Shibl, 2015).

In this study, LA and LA-ET were both shown to exclude EHEC O157 from LoVo cells in exclusion and competition assays, which highlights the important role of probiotics in interrupting progressive infection with EHEC O157:H7. This finding was consistent with studies in which probiotics reduced intestinal infection (Resta-Lenert and Barrett, 2003; Rodrigues et al., 2012). Furthermore, the FAS test showed that LA-ET has the ability to inhibit A/E lesions. It is obvious that LA-ET inhibits EHEC O157:H7 to induce the formation of A/E lesions and adhesion to cells. However, recombinant anti-EspA antibodies block EHEC O157:H7-induced A/E lesions *in vitro* but do not affect EHEC O157:H7 adhesion to host cells (La Ragione et al., 2006). Therefore, the effect of LA-ET in excluding EHEC O157:H7 from adhering to LoVo cells may be attributed to the probiotics competing with pathogens for binding sites.

LA-ET used for oral immunization can prevent gastric infection and allows direct contact between the antigen and the immune system. Significant IgG and sIgA responses were elicited in mice immunized with LA-ET and protective effects were also confirmed. A previous study showed that attenuated EIS-producing recombinant *Salmonella* induced significant specific IgG in serum and sIgA in feces to protect mice from a challenge of EHEC O157:H7 (Gu et al., 2011). These findings indicate that antibody-mediated immunity participates in the prevention of EHEC O157:H7 infection. In addition, the rate of protection was consistent with antibody and cytokine levels in vaccinated mice. LAB strains for vaccines can be specifically selected according to their function to modulate dendritic cells and induce Th1, Th2, or mixed Th1/Th2 responses (Wells, 2011). The major cytokines associated with Th1 cells are IL-2, TNF-α and IFN-γ, which can enhance T cell cytotoxicity and immune responses. Th2 cells secrete IL-4, IL-5, and IL-10, which mainly promote antibody production and mediate humoral immune responses (Zhu et al., 2016). We observed that LA-ET enhanced the production of IFN-γ, IL-4, and IL-10, indicating that a mixed Th1/Th2 response was induced. Thus, immunizing mice with LA-ET induced humoral and cellular immunity, consistent with previous findings (Ahmed et al., 2014).

Reduced intestinal colonization is regarded as an important criterion for protection against EHEC O157:H7 infection. Oral immunization of mice with LA-ET resulted in a reduction of EHEC O157:H7 fecal shedding in two infection models. The infection model under the oral streptomycin treatment condition emphasized the protective effects of humoral and cellular immune responses induced by LA-ET. LA-ET blocks adhesion of EHEC O157:H7 to intestinal tract, which may be associated with the sIgA. The sIgA antibodies play an important role in blocking the attachment of EHEC O157:H7 to epithelial cells (Ghaem-Maghami et al., 2001). The infection model without streptomycin treatment took into account the probiotic properties of LA-ET. Probiotics have been shown to protect mice against EHEC O157:H7 (Shu and Gill, 2002). In this study, we found that LA elevated IL-10 secretion, reduced EHEC O157:H7 colonization (without streptomycin treatment), and increased the survival rate in mice, suggesting that *L. acidophilus* strains play an important role in protecting mice against EHEC O157 infection and may be useful vaccine carriers for combating enteric pathogens in humans.

The histopathology indicates that administration of LA-ET has the ability to reduce or inhibit A/E lesions, which suggests that LA-ET could reduce the colonization of EHEC O157:H7 in the intestinal tract and inhibit A/E lesions. Moreover, the mechanism utilized by LA-ET against EHEC O157:H7 is different from that of antibiotics. LA-ET was safe in a cell model, but EHEC O157:H7 released toxins when treated with antibiotics, which could cause toxin-induced systemic injury. Thus, LA-ET is a promising candidate vaccine against EHEC O157:H7, especially for children and the elderly. Although oral immunization with LA-ET induced protective immunity, an important deficiency should be noted. First, the antigen dose provided by LA-ET *in vivo* cannot be measured. In addition, the expression level of the antigen in LAB is weak and the enzymes in the gastrointestinal tract may digest the proteins (Guo et al., 2015), which may reduce the effects of antigen. Furthermore, Ahmed et al. (2013) previously constructed a recombinant *L. lactis* strain expressing the EHEC antigen EspB and this strain expressed rather low levels of antigen EspB. However, they optimized the expression of EspB in *L. lactis* through secretion of EspB either under constitutive or nisin-inducible control, and the strains successfully induced immune responses (Ahmed et al., 2014). Collectively, we speculated that an oral vaccine would be more effective, than LAB vaccines that are engineered to co-express antigens with cytokines or made inducible in different mutants, or new strains that are selected to have enhanced adjuvant or targeting capacities (Rosales-Mendoza et al., 2016). In our study, the antigen expressed by LA-ET was not strong, which was shown by SDS-PAGE analysis. This is the first report of the use of *L. acidophilus* expressing the EHEC O157:H7 antigen. Thus, the optimal condition for LA-ET that can enhance stimulation of strong immune responses still needs to be explored. High expression of *L. lactis* vector is promising for vaccine development.

## Conclusion

LA-ET was capable of inhibiting A/E lesions and bacterial adhesion *in vitro*, and oral immunization strain induced both humoral and cellular immune responses *in vivo* and protected against EHEC O157:H7 colonization and infection in mice. These findings suggest that oral LA-ET is a promising candidate vaccine against EHEC O157:H7 infection.

## Author Contributions

RL did the experiments with gene construction, cells and mice, analyzed data, and wrote the manuscript. YZ did the experiments with mice and analyzed data. BL designed the experiments and contributed to revising the manuscript. YL and YW did the experiments with gene construction and mice. SD and BZ did the experiments with mice. XW and HF provided overall directions and contributed to revising the manuscript.

## Conflict of Interest Statement

The authors declare that the research was conducted in the absence of any commercial or financial relationships that could be construed as a potential conflict of interest.
